# Filling Teeth

**Published:** 1857-06

**Authors:** R. Arthur


### FILLING TEETH.

There is no subject connected with dentistry about which so much has been said and written as about the operation of "filling" or "plugging" the teeth. In all conversations between dentists, this is a topic which never fails to furnish points of interest. There is scarcely a number of a professional periodical issued which does not contain some article or part of an article devoted to it. It has not, however, received an undue amount of attention. We all recognize the fact that, in the thorough performance of this operation, lies the greatest benefit the dentist is able to render to those who, unfortunately, find it necessary to place themselves in his hands. As its object is to arrest the progress of that disease which leads to nearly all the trouble he is called upon to remove, it may well be regarded as the great remedial measure of his art. Although, as I have said, this subject has been discussed in a great variety of bearings, it is far from being exhausted.

There is one feature of this operation which has been regarded by many writers and by many operators as a cardinal point; it is this: that the filling is most nearly perfect when it is made up of gold (as this is generally conceded to be the best substance known for the purpose) in its most compact state. The more nearly it is brought into the condition of gold that has been fused, the more perfect the filling is regarded.

It is conceded that the object of filling teeth is to place in the cavity formed—as a consequence of the decomposition and removal of a portion of the dentine, occasioned by the chemically solvent properties of the fluids of the mouth, or some agent contained or generated therein—a substance capable of resisting this solvent action. The material used must not only be possessed of the property referred to, but it must be so placed in the cavity as to be absolutely impermeable to the fluids of the mouth; must be brought into such perfect contact with the walls and orifice of the cavity as to prevent the passage of any fluid alongside of it. Where the cavity is situated on a grinding surface, the filling must also be capable of resisting the pressure and friction of mastication.

Though far from advocating hasty and slovenly operations, of which, I think, I shall not be accused by those familiar with what I have done in this way, I have little hesitation in asserting that a great deal more stress has been laid upon this feature of filling the teeth than it deserves.

A certain degree of density is essential, but this, I am well convinced, is far short of the degree of density which may be obtained. I have very little question that gold foil may be made quite as compact as gold melted and poured into a cavity of exactly the same dimensions as the one filled, but this I regard as, in any case, useless.

I will state explicitly what I mean. If I have, for example, a large cavity situated so as to be accessible, in filling which I can use direct force to advantage, I am well satisfied that, after getting a sufficient quantity of annealed gold condensed so as to retain its place, I can take pellets, made up of a quarter of a sheet of No. 6 gold each, and condense with an instrument, the point of which shall not be less than one line in diameter, using as much force as I can bring to bear upon it, and when I have completed the operation it will be dense enough to answer all the purposes of a filling. If it is brought into good contact with all the sides of the orifice of the cavity, and this last has been well prepared, and afterward the whole surface of the filling is well polished, I should expect such a filling to preserve the tooth in which it was put as well as any other filling; and yet I could put in the same cavity at least one-eighth more gold.*

This, indeed, is not a matter that need rest upon my own statement. Every operator must acknowledge that there is necessarily considerable variation in the density of different fillings in the same mouth. In some cases it is an absolute impossibility to bring the same amount of pressure to bear upon the gold as in some others. And there are cases, I mean those of frail teeth, in which it is quite impossible, particularly under the old system of using gold, to make a filling anything like so dense as it can be made where the walls of the cavity are strong. And yet, if all the other conditions of a good filling are observed: if the orifice of the cavity has

---

* In using "annealed gold," as I have been doing almost exclusively since my first communication to the "News Letter" on this subject, a number of improvements in the methods of manipulating with it have suggested themselves. A pellet of a quarter of a sheet of No. 6 foil, if made in the ordinary way, that is, by rolling a sheet into a rope, annealing it, and cutting it into four pellets, would be nearly unmanageable; it could not be well condensed with instruments of any size or form. A better method of making the pellets is the following: The sheet, without being folded or rolled up, is annealed; it is then cut or torn into pieces of the size desired, and drawn together with the instruments used for plugging. In this way, large pellets can be made as loose as small ones; any degree of density, indeed, may be given to them. When the gold is prepared in this way, instruments with a larger condensing surface may be employed; it may be worked more rapidly and made denser than where a closer pellet is used.

With regard to the use of "annealed gold," I cannot avoid taking occasion here to say, that I am more than ever satisfied that it is by far the best method of using gold for this purpose, of which I have any knowledge. I feel confident, that in time it will become the nearly universal method of using foil for filling teeth, unless some better method, of which nothing is now known, is devised. I find its advantages so great, and the same advantages have become apparent to a number of good operators with whom I am acquainted, that I am well convinced that its use is not fully understood by those who reject it; or at least that they have not learned to overcome the slight difficulties sometimes attending its use in the beginning.

been well prepared; if the gold has been brought into intimate contact with all parts of the walls and orifice, and if the surface has been well finished, we do not look for failure merely because the gold is not so dense as it might have been made.

It is my decided impression, that gold cannot be made so dense, by a great deal, when it is used in the form of cylinders, as it can be by other methods, even in the hands of those most successful in its use. And yet I cannot but look upon this method of filling teeth as excellent, and its excellency, in my estimation, consists in the very perfect contact which can be obtained between the gold and the walls of the cavity filled in this way.

There is no dentist, I presume, of a few years' practice, who has not met with fillings which have completely arrested decay, so soft that, with a very slight effort, an instrument could be thrust through them; so soft as to make it impossible to remove them without tearing them to pieces. And yet such fillings, as I have said, have preserved the teeth for years.

I presume I can scarcely be misunderstood. It is scarcely necessary to say that a good filling should be a dense one. No operator who fills teeth, in the manner just indicated, although he may be successful in some cases, can ever become a good operator. Nothing will justify a dentist in operating carelessly. But in order to fill teeth well, a great deal of labor and time and care are necessary. It is well, therefore, to consider how much time and labor are absolutely required, so that neither may be wasted.

These are, of course, mere hints in this direction. It is a subject, however, I am satisfied, of importance, and well worthy the careful attention of every practicing dentist.*—*Dental News Letter.* R. ARTHUR.

---

* As I have adopted exclusively the use of "annealed gold" in my practice, and have strongly urged its advantages, it may, possibly, be inferred from the above article, that I admit the truth of the assertion, that it is impossible to force as much gold into a given cavity, if it possessed the adhesive qualities necessary to its use, as I have proposed, as if it has the ordinary character of gold foil. I have no hesitation in saying, and my confidence is based not only upon the plain reason of the thing, but upon some recent experiments in this direction, that I can put as much "annealed gold into a given cavity, in the same time, with the use of the same amount of force, as can be done by any other method at present known.

